# Hooking the big one: the potential of zebrafish xenotransplantation to reform cancer drug screening in the genomic era

**DOI:** 10.1242/dmm.015784

**Published:** 2014-07

**Authors:** Chansey J. Veinotte, Graham Dellaire, Jason N. Berman

**Affiliations:** 1 Department of Pediatrics, IWK Health Centre, PO Box 9700, 5850/5980 University Avenue, Halifax, NS, B3K 6R8, Canada.; 2 Life Sciences Research Institute, Faculty of Medicine, Dalhousie University, 1348 Summer Street, Halifax, NS, B3H 4R2, Canada.; 3 Department of Pathology, Dalhousie University, Sir Charles Tupper Medical Building, 5850 College Street, Halifax, NS, B3H 4R2.; 4 Department of Microbiology and Immunology, Dalhousie University, Sir Charles Tupper Medical Building, 5850 College Street, Halifax, NS, B3H 4R2, Canada.

**Keywords:** Cancer, Drug screening, Microenvironment, Xenotransplantation, Zebrafish

## Abstract

The current preclinical pipeline for drug discovery can be cumbersome and costly, which limits the number of compounds that can effectively be transitioned to use as therapies. Chemical screens in zebrafish have uncovered new uses for existing drugs and identified promising new compounds from large libraries. Xenotransplantation of human cancer cells into zebrafish embryos builds on this work and enables direct evaluation of patient-derived tumor specimens *in vivo* in a rapid and cost-effective manner. The short time frame needed for xenotransplantation studies means that the zebrafish can serve as an early preclinical drug screening tool and can also help personalize cancer therapy by providing real-time data on the response of the human cells to treatment. In this Review, we summarize the use of zebrafish embryos in drug screening and highlight the potential for xenotransplantation approaches to be adopted as a preclinical tool to identify and prioritize therapies for further clinical evaluation. We also discuss some of the limitations of using zebrafish xenografts and the benefits of using them in concert with murine xenografts in drug optimization.

## Introduction

The zebrafish is a powerful model system for studying human cancer. The ease with which these organisms can be genetically manipulated, the opportunity for direct observation in transparent embryos, and the capacity for forward genetic and chemical screens has enabled zebrafish to be employed in the investigation of many gain-of-function and loss-of-function mutations implicated in cancer (Amatruda et al., 2002; Beckman, 2007; Beckwith et al., 2000; Berghmans et al., 2005; Dovey et al., 2009; Gutierrez et al., 2011; Yang et al., 2004). Many of the oncogenes and tumor suppressor genes that have been identified as major players in human cancers have zebrafish homologs, and important signaling pathways regulating cell proliferation, migration, apoptosis and differentiation are well conserved (Feitsma and Cuppen, 2008; Payne and Look, 2009; Stoletov and Klemke, 2008). In parallel with the basic characterization of important cancer-related genes and pathways in the zebrafish, a toolkit of experimental approaches has also been developed that has enabled cancer modeling in this species. This toolkit includes many reverse genetic approaches to generate loss-of-function phenotypes, such as TILLING (targeting induced local lesions in genomes) (Wienholds et al., 2003), morpholino oligonucleotides (Nasevicius and Ekker, 2000), and more recently, zinc-finger nucleases (Doyon et al., 2008), TALENs (transcription activator-like effector nucleases) (Huang et al., 2011) and the CRISPR (clustered regularly interspaced short palindromic repeats) system (Hwang et al., 2013). Gain-of-function approaches have also been developed, including transgenic techniques to introduce foreign oncogenes into the genome, which can incorporate meganucleases (Soroldoni et al., 2009), Tol2 transposons (Kwan et al., 2007) or bacterial artificial chromosomes (BACs) (Suster et al., 2011). Through these approaches, numerous mutant and transgenic zebrafish have been generated to model human cancers, and these have also been used in chemical screens. In addition to these cancer models, a number of mutant and transgenic zebrafish lines have been generated that facilitate the observation of oncogenic pathways in living fish, such as the *casper* mutant (White et al., 2008), a transparent zebrafish strain that maintains embryonic transparency through to adulthood because it lacks melanocyte and iridophore cell populations, and the vascular fluorescent reporter line *tg(fli1a:eGFP)* (Lawson and Weinstein, 2002). Together with advances in imaging technology, these lines have provided new opportunities to develop the zebrafish as a xenograft model with potential for *in vivo* drug screening.

Xenotransplantation is the transfer of living cells or tissue from one species to another (Cariati et al., 2011). The appeal of zebrafish xenotransplantation of human cancers for drug discovery and evaluation lies in the ability to directly observe the drug response of human tumor material, particularly primary patient-derived biopsy specimens that are often hard to maintain *in vitro*, in a cost-effective and time-efficient manner. The approach circumvents some of the current hurdles with rodent xenografts, such as those related to availability of sufficient patient material or challenges in optimizing murine hosts to enhance engraftment. Moreover, zebrafish xenotransplantation approaches have the potential to serve as a first step in a preclinical pipeline to screen for drugs before moving on to the more costly and time-consuming mouse and ultimately human trials. The zebrafish xenograft platform could also, for the first time, provide a bona fide real-time *in vivo* platform for personalizing cancer therapeutics.

### Small-molecule drug screens in zebrafish

Pioneering work undertaken by several laboratories has demonstrated the efficiency of large-scale drug screens in zebrafish embryos. Arguably, these studies and the therapeutic revelations that have emerged from them represent some of the most clinically relevant contributions the zebrafish model has made in the last decade (Tamplin et al., 2012; Zon and Peterson, 2005). One of the first chemical screens using zebrafish embryos was piloted by Randall Peterson’s group (Peterson et al., 2000). They conducted a screen using a panel of structurally diverse chemical compounds and evaluated toxicity in embryonic development, including on the central nervous and cardiovascular systems. The authors identified several compounds that influenced the development of the central nervous system, altering its general morphology by significantly increasing the size of the hindbrain ventricle and producing tissue artifacts such as ‘sawtooth-like’ projections within the organ (Peterson et al., 2000). Subsequently a number of studies have demonstrated the utility of chemical screening in zebrafish embryos for identifying and evaluating the efficacy of potential anti-cancer agents (Murphey et al., 2006; Ridges et al., 2012; Yeh et al., 2009).

## Technical considerations

Zebrafish are ideally suited to high- and medium-throughput screens because their small size enables them to be arrayed in a variety of isolated well plates (12-well, 24-well, 96-well and larger) and bathed in water that contains the compound(s) of interest (reviewed in Peterson and Macrae, 2012). This approach provides a high-throughput platform that is substantially more rapid than injecting mice. Furthermore, embryo screens have the potential to reveal important information on absorption, distribution, metabolism and excretion when whole organisms are exposed to a drug (Makky et al., 2008; Peterson and Macrae, 2012). However, investigating these properties in embryo screens is still in its infancy. Zebrafish embryos are transparent and develop externally, which can aid in understanding drug absorption from the surrounding medium specifically when chemicals possess inherent fluorescence to facilitate direct visualization of drug absorption into the embryo. Subsequent drug excretion following treatment can also be observed and measured by exploiting the transparency of zebrafish embryos. However, this approach requires that compounds are water-soluble. Thus, drug solubility characteristics need to be established at the outset, including maximum water solubility or drug solubility in other common delivery solvents, such as dimethyl sulfoxide (DMSO) or methanol, before undertaking these types of screens. If compounds are not water-soluble, direct injection into the body of the embryo can be performed to ensure drug uptake. Both diffusion and injection approaches can be used for toxicity screens and to evaluate specific efficacy signals.

## Evaluating drug toxicity

Systemic drug toxicity can be investigated using overall embryonic mortality as a metric from which the working range of a particular drug can be determined. For example, embryos (one or more per well) can be arrayed in multi-well plates and exposed to a series of drug concentrations to enable the maximum tolerated dose of a compound to be determined (Parng et al., 2002; Taylor et al., 2010). These studies can uncover toxic side effects within the context of a living animal that are not discernible in tissue culture. In this way, appropriate dosing can be determined prior to performing a chemical screen to evaluate drug efficacy in order to ensure that observations are not confounded by non-specific drug toxicity.

Candidate compounds from *in vitro* tissue culture screens can also be further evaluated using the zebrafish. Precise phenotypic observations can be made to determine drug-induced effects on specific aspects of development and tissue function. For example, the development of pericardial edema, changes in specific tissue or cell types made fluorescent through versatile transgenic strains, or altered growth of different organ systems (Barros et al., 2008; Eimon and Rubinstein, 2009; Sipes et al., 2011). For instance, a compound that selectively kills cancer cells in the tissue culture dish but that also causes severe central nervous system toxicity or renal damage *in vivo* might not be a good candidate drug for further development.

## Applications of drug screening in blood diseases and angiogenesis

Zebrafish screens have made important contributions to the study of both normal and leukemic hematopoiesis. A chemical screen in wild-type embryos conducted by North, Zon and colleagues in 2007 (Durand and Zon, 2010; North et al., 2007) revealed a previously unrecognized role for prostaglandin E2 (PGE2) in the development of hematopoietic stem cells (HSCs). Using whole-mount *in situ* hybridization for increased expression of the HSC markers *runx1* and *c-myb*, ten of the effective compounds were found to modulate the prostaglandin pathway. This discovery was subsequently validated in mice and led to a recent clinical trial (Cutler et al., 2013), suggesting that the use of PGE2 can enhance HSC engraftment following cord blood transplantation. This finding represents the first time a chemical screen in zebrafish has led to a clinical trial in patients and highlights the tremendous potential of embryonic screens for drug discovery. In another study (Yeh et al., 2009), the transgenic zebrafish strain *tg(hsp:AML-ETO)* was used to screen for chemical modifiers of AML1-ETO, an oncogenic fusion protein that is prevalent in acute myeloid leukemia (AML) that results in hematopoietic dysregulation in fish and elicits a malignant phenotype similar to human AML. An inhibitor of cyclo-oxygenase 2 (COX-2), nimesulide, was identified as an antagonist to AML1-ETO in hematopoietic differentiation. Furthermore, this screen exposed a role for COX-2 and PGE2 in the subsequent phenotypes associated with *AML1-ETO* expression (Zhang et al., 2013).

Other zebrafish drug screens have been designed to identify agents targeting tumor angiogenesis. Angiogenesis is a physiological process in which normal blood vessels are formed during embryonic development to deliver oxygen and nutrients to the required tissues of the body. Tumor cells have been shown to co-opt this process and use angiogenic recruitment for cell growth and subsequent metastasis (Camus et al., 2012; Chan et al., 2002; Cross et al., 2003; Radi et al., 2012; Tran et al., 2007). Many of these studies have used a non-cancer model, the *tg(fli1a:eGFP)* zebrafish strain, in which the *fli1a* promoter drives the expression of green fluorescent protein (GFP) in embryonic blood vessel endothelial cells, as a surrogate assay (Lawson and Weinstein, 2002). Common readouts in zebrafish drug screens have included changes to local blood vessel and hematopoietic cell organization and formation, alterations of organ development such as the heart and kidneys, and behavior changes such as swimming and reaction responses, following exposure to a chemical agent (Burns et al., 2005; Cao et al., 2009; Kokel et al., 2010; Murphey et al., 2006; North et al., 2007; Paik et al., 2010; Peterson et al., 2004; Ridges et al., 2012; Stern et al., 2005; Yeh et al., 2009).

As highlighted above, some of the most successful zebrafish screens have been in the identification of a new application for an existing drug, so-called drug repurposing. This is advantageous when familiarity and available human safety data can permit rapid transition to the clinical setting, as illustrated by the prostaglandin example discussed above (Durand and Zon, 2010). However, new drugs are also being identified in this way. For example, Ridges et al. (Ridges et al., 2012) recently applied a chemical screening strategy to a panel of T-cell acute lymphoblastic leukemia (T-ALL) mutant embryos and identified lenaldekar, a new compound, as a selective inhibitor of lymphocyte proliferation. Over 26,000 compounds were screened for activity that could reduce overall fluorescent-tagged lymphoblast populations in mutant embryos. Lenaldekar was shown to induce remission in a zebrafish T-ALL model without causing substantial toxicity. Similar observations and drug efficacy was confirmed using murine xenograft models (Ridges et al., 2012). These studies underline the versatility and robustness of using the zebrafish in small-molecule chemical screens with similar experimental design but diverse goals. As pharmaceutical companies increasingly make available their substantial chemical library resources to academia, there is no doubt more such discoveries will be made, and the zebrafish will be prominent in their screening.

In summary, drug screens in zebrafish have been used for and have the potential to validate existing compounds for use in many human disorders. In drug discovery studies, promising new compounds can be identified from panels of thousands in smaller quantities than in rodent studies, which require large volumes of reagents. Zebrafish embryos can be used to identify general drug toxicity and gross developmental abnormalities caused by drugs and to prioritize agents that should be further evaluated in additional preclinical model systems.

Human tumor xenografts in zebrafish are an innovative approach that can take full advantage of the inherent opportunities afforded by the zebrafish but with the added benefit of addressing any lack of conservation in drug response or molecular oncogenic pathways between humans and fish, and zebrafish xenografts are gaining traction for advancing cancer drug development.

## Zebrafish xenotransplantation

Transgenesis has been a traditional approach to modeling human malignancies in zebrafish (Carradice and Lieschke, 2008; Chen and Langenau, 2011; Forrester et al., 2011; Jessen et al., 1999; Le et al., 2007; Leacock et al., 2012; Li et al., 2013; Project et al., 2005) but, more recently, the xenotransplantation of human cancer cell lines and primary human tumor samples has been successfully used to study human cancers *in vivo* (Corkery et al., 2011; Eguiara et al., 2011; He et al., 2012; Jung et al., 2012; Konantz et al., 2012; Nicoli et al., 2007; Taylor and Zon, 2009).

Xenotransplantation involves the transfer of one species-specific tissue to another animal species and has been used as a tool for many years to study human cancer (Cariati et al., 2011; Johnson et al., 1995; Merk and Adams, 1972). In particular, xenograft experiments have been extensively performed in mouse models of human disease, enabling the analysis of cancer cell proliferation, invasion, migration and induction of growth advantages such as increased angiogenic recruitment (Cheon and Orsulic, 2011), as well as enabling drug screening and evaluation (Huynh et al., 2011; Kelland, 2004; Sharpless and Depinho, 2006). Although murine models remain the ‘gold standard’ for xenotransplantation studies and drug evaluation, the time required, high cost and complexity of these systems mean that it is not always feasible to use mice. This is particularly relevant when funds to conduct such large-scale drug screens are limited. Moreover, the use of highly immunecompromised mice calls into question the actual relevance of findings in these models back to the bedside. By contrast, the ease and relative cost-effectiveness of performing these screens in zebrafish can be adapted to embryos transplanted with human cancer cells without the need for immunosuppression because, for the first month of life, the larvae have not developed an adaptive immune response (Lam et al., 2004; Traver et al., 2003).

The zebrafish xenotransplantation model can also be applied to drug screening and rivals transgenic approaches by allowing the direct evaluation of the most clinically relevant tissue, namely human cell lines or primary samples. This circumvents the limitations imposed by the lack of zebrafish-specific tools, such as antibodies for flow cytometry or for western blot confirmation, and the apparent absence of certain key genes that regulate human oncogenic pathways, such as the *BRCA1* and *INK4a/ARF* tumor suppressors (Howe et al., 2013). The absence of certain genes in the zebrafish can also be exploited as a means of identifying human xenografted cells from the zebrafish embryo in *ex vivo* analyses and immunohistochemistry. For example, we have recently used the promyelocytic leukemia (PML) protein, for which the gene is absent in the zebrafish, as a marker of human primary T-cell leukemia cells during *ex vivo* proliferation analyses (Bentley et al., 2013). Finally, it should be noted that the human tumor xenografts interact with, and in many instances remodel, the host tissue following engraftment; for example, host vasculature can be recruited by tumor xenografts (Zhao et al., 2011). This is a useful property of the xenotransplantation system in zebrafish, allowing analysis of tissue remodeling, innate immune cell interactions with the human tumor and neoangiogenesis (tumor-associated angiogenesis) induced by the human tumor. These changes to the host might lead to developmental toxicities, precluding analysis of tumor progression. For this reason, all zebrafish xenotransplantation experiments begin with pilot experiments to ensure that the given tumor being studied can successfully engraft and that the zebrafish embryo can survive engraftment long enough to allow meaningful observations to be made.

## Technical advances in zebrafish xenotransplantation

Lee et al. (Lee et al., 2005) first pioneered human tumor xenotransplantation in zebrafish by engrafting human melanoma cell lines, demonstrating that human cells could survive and migrate in zebrafish. Melanoma cells were injected into the blastula stage of zebrafish embryos. These cells were tolerated within the zebrafish for the first 8 days after injection and could home to the epidermis. Human fibroblasts and normal healthy melanocytes were also injected into zebrafish and displayed similar survivability to that of the melanoma cells, but lacked the mobility of the latter. Further studies have developed and optimized the parameters for transplanting human cancer cell lines, including breast cancer, leukemia and sarcomas, into zebrafish embryos and analyzing the transplanted recipients for cellular behavior and response to drugs (Corkery et al., 2011; Pruvot et al., 2011; Haldi et al., 2006; Marques et al., 2009). Human cancer cell lines survived, formed tumors and exhibited cell migration in zebrafish embryos.

Haldi et al. optimized transplantation parameters such as: site of injection, age of transplant recipients, injection cell quantity and post-injection incubation considerations (Haldi et al., 2006). Although numerous sites of injection were trialed, including the hindbrain ventricle and intravenous routes, the yolk sac of 2-day-old embryos was demonstrated to be an ideal site of injection because it provides a nutrient-rich environment for injected cells and is acellular, allowing cell growth and migration (achievable only through active cell motility) to be monitored. This group also showed that 35°C is the optimal temperature at which to incubate embryos following xenotransplantation. This temperature permits both natural embryonic development of the zebrafish transplant recipients and satisfactory human cell survival and proliferation. Marques et al. expanded on the field of zebrafish xenotransplantation by qualitatively capturing hallmark oncogenic events of human cancer cells post-embryonic injection (Marques et al., 2009). Patient-derived gastrointestinal tumor cells displayed cell proliferation, migration through the circulation and micrometastases within 24 hours following transplantation – events that were not observed using non-tumor cell lines (Marques et al., 2009). Injected pancreatic tumor cells transplanted into the *cloche* zebrafish mutant, which lacks a complete functional vasculature, displayed no cell migration (Herpers et al., 2008). These observations demonstrate that migration observed post-xenotransplantation of human cell lines into zebrafish embryos occurs via active transport mechanisms such as a circulatory route, and not by diffusion or other passive means.

With the establishment of these foundational parameters, xenotransplantation methods have continued to improve, with the incorporation of single-cell-based assays to quantitatively interpret *in vivo* human cell behavior. We have established approaches to reproducibly quantify cell proliferation rates and cell migration in zebrafish xenotransplantation, a necessity for applying xenotransplantation to determining drug responses (Corkery et al., 2011). Cancer cells are fluorescently labeled and injected into the embryos, and cell proliferation is monitored by live-cell microscopy. Embryos are then enzymatically dissociated into cells, which are stained to confirm that they are intact and then imaged to determine average cell counts per embryo (Corkery et al., 2011). Proliferation rates of several cell lines, including leukemia, sarcoma and breast cancer lines, have been measured using this assay, and proliferation rates of cells injected in zebrafish embryos demonstrate similar kinetics to those observed *in vitro*. When fluorescence cannot be used to mark human cells either because the cells are dividing too rapidly or the drugs being tested are inherently fluorescent (e.g. doxorubin), an immunocytochemical technique can be applied that uses a fluorescent antibody directed against a human protein not conserved in zebrafish (Bentley et al., 2013) (V. L. Bentley, C.J.V., D. P. Corkery, J. B. Pinder, M. A. LeBlanc, K. Bedard et al., unpublished results). Using these approaches, we have demonstrated the utility of zebrafish xenotransplantation for the study of specific drug-tumor interactions for both chronic myelogenous leukemia and acute promyelocytic leukemia by specifically inhibiting their proliferation in xenotransplanted zebrafish using imatinib mesylate or all-*trans* retinoic acid, respectively (Corkery et al., 2011). We have also utilized the zebrafish xenotransplantation assay to evaluate transcription factors that may be critical for migration and metastasis in childhood sarcomas (Veinotte et al., 2012) (A. M. El-Naggar, C.J.V., C. E. Tognon, D. P. Corkery, C. Hongwei, F. Tirode et al., unpublished results).

Many zebrafish xenotransplantation studies performed to date have used embryos at 48 hours post-fertilization (hpf) as transplant recipients. This developmental time point is considered the optimal stage for injection because it offers a relatively large transplant site (yolk sac) and absence of adaptive immune responses. Immunosuppression is required for xenotransplantation in zebrafish older than 30 days post-fertilization (dpf), when adaptive immunity is well-established (Lam et al., 2004). Other advantages that are apparent in transplanting human cancer cells into 2- to 5-day-old (48–120 hpf) embryos include the large number of animals that can be produced for experimentation, injected and housed simultaneously.

**Fig. 1.**
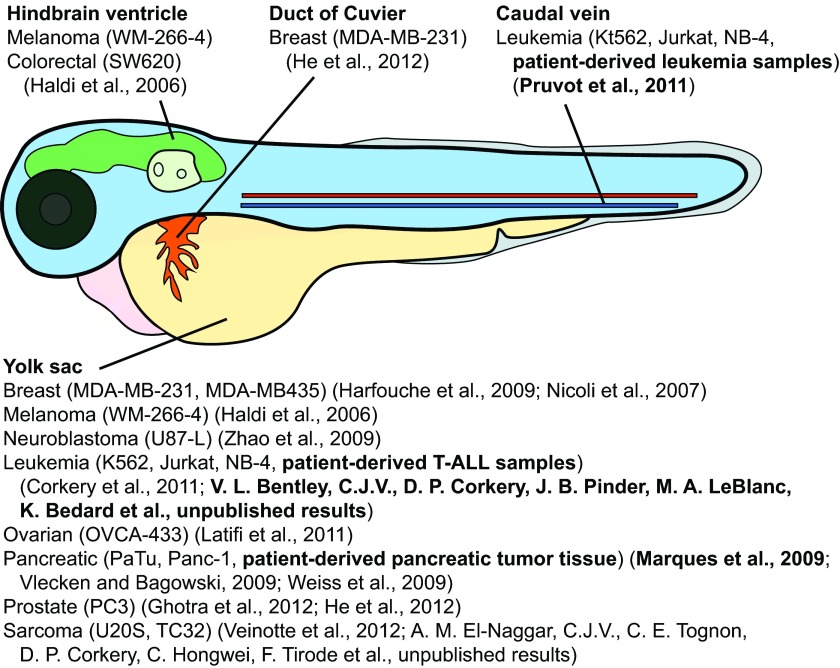
**The most common injection sites used in xenotransplantation of 48 hpf zebrafish.** The yolk sac is the most frequently used site of injection. Several human cancer cell lines (indicated in parentheses) and primary cancer samples (indicated in bold text, as well as their accompanying references) have been transplanted into various anatomic sites.

The yolk sac has been considered the optimal site of transplantation because it provides a favorable acellular site of injection that can nurture a variety of human cancer cell lines prior to and/or during tumor growth and cell migration. Other anatomical locations have also been used (Fig. 1), although the yolk sac remains the most widely used site of embryonic xenograft injection to date. The site selected can influence how a tissue sample engrafts, the efficiency of drug administration and delivery to tumor-engrafted materials, and, subsequently, response to therapy. Many of the sites used for injection are not native to the tumor tissue, so heterotopic engraftment is necessary. Although the zebrafish shares homology with many of the mammalian pathways and organ systems, it does lack several tissues found exclusively in mammals, which affects the possibility of orthotopic injection. Despite the lack of orthotopic sites for tissues such as the lung, breast and prostate, it might be possible to ‘add-back’ the required cells or growth signals that would allow normal growth cues associated with orthotopic injection. For example, embryo water could be supplemented with tissue-specific growth factors (e.g. androgens or estrogens), human cells expressing growth factors could be co-injected with the cells of interest, or transgenic fish expressing human growth factors, receptors and/or cytokines could be generated to enhance engraftment and tumor growth.

## Zebrafish xenotransplantation as a tool to study tumor-microenvironment interactions

Another opportunity afforded by zebrafish xenotransplantation is that it enables direct observation of the impact of a particular treatment on interactions between tumor cells and the tumor microenvironment. A number of studies have highlighted the impact of the tumor microenvironment on the proliferation and migration of a variety of human cancers (Gupta and Massagué, 2006; Hanahan et al., 2000; Yu and Rak, 2003). Many normal cell and/or tissue behaviors are often co-opted by and for malignant cells. Solid tumor cells harness the oxygen and nutrients needed for their survival from oncogenic-induced blood vessels sprouting from healthy normal vasculature (Kaya et al., 2009; Singh et al., 2007; Zetter, 1998). Immune cells that normally combat infection might function in either pro-tumorigenic or anti-tumorigenic ways depending on cytokines and chemokines released by tumor cells and other inflammatory mediators generated in the local microenvironment (Kees and Egeblad, 2011; Spano et al., 2012). Studying and uncovering the interactions that take place between tumor and normal cell populations is essential for understanding cancer progression and for determining new ways to therapeutically inhibit pro-tumorigenic responses and enhance anti-tumorigenic behavior.

**Fig. 2.**
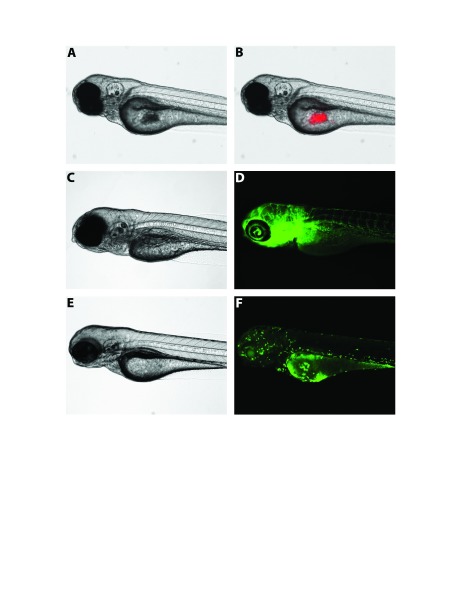
Transgenic zebrafish can be used concurrently with xenotransplantation models to study the interactions between injected human cancer cells and fundamental elements of the host microenvironment. Panel A shows a brightfield image of a 4 dpf *casper* embryo (White et al., 2008), and panel B, a fluorescence image, shows the same embryo with injected Cm-Dil-labeled TC32 Ewing’s sarcoma cells (in red). Panel C, a brightfield image, shows an uninjected 5 dpf *fli1a:eGFP* (Lawson and Weinstein, 2002) *casper* embryo, and panel D, a fluorescence image, shows the same embryo with GFP-labeled vasculature. Panel E is a brightfield image showing an uninjected 4 dpf *mpeg1:eGFP* (Ellett et al., 2011) *casper* embryo, and panel F, a fluorescence image, shows the same embryo with GFP-labeled monocytic cell lineages, including macrophages. dpf, days post-fertilization; GFP, green fluorescent protein.

Fluorescent transgenic zebrafish reporter lines represent a key tool that can be paired with xenotransplantation to study cancer cell progression in a living microenvironment (Fig. 2). Currently available fluorescent reporter lines include *tg(fli1a-eGFP)* (Lawson and Weinstein, 2002), which expresses enhanced GFP (eGFP) in endothelial cells; *tg(mpx:eGFP)*, which expresses GFP in neutrophils (He et al., 2012; Renshaw et al., 2006); and *tg(mpeg1:eGFP)* (Ellett et al., 2011), which expresses GFP in monocytes/macrophages. Combining such transgenic strains of zebrafish with transparent strains, such as the *casper* mutant, allows for the observation and analysis of tumor cell interactions with key players in the tumor microenvironment.

As mentioned above, there is a large time lapse of a number of weeks between the development of innate and adaptive immune systems in the zebrafish embryo. This temporal separation in the development of neutrophils, macrophages/monocytes and mast cells, and subsequently B and T cells, provides a unique opportunity to evaluate the specific contribution of each of these lineages to cancer progression and therapeutic response (He et al., 2012). He et al. showed that neutrophil migration could control tumor cell mobility within zebrafish embryos through pre-conditioning of extracellular matrix, resulting in the formation of metastatic niches. The group also showed that vascular endothelial growth factor receptor inhibitors reduce local tumor growth of xenografted embryos and blood vessel recruitment to the tumor site. These compounds also enhanced the rate of neutrophil migration, which in turn increased the migratory potentially of the injected tumor cells, resulting in micrometastases. These observations demonstrate how zebrafish xenotransplantation can be used to study tumor cell and tumor microenvironment interactions, validate drug efficacy of compounds that directly target elements of the microenvironment to prevent cancer progression, and uncover any unwanted or harmful bystander cell effects prior to moving a compound further along the preclinical pipeline.

## Limitations of the zebrafish xenotransplantation platform for drug screening

Although all of the characteristics described above enable the completion of multiple statistically robust experiments simultaneously, the zebrafish xenotransplantation platform is not without its limitations. Although the lack of an adaptive immune response is beneficial for initial transplantation and injection, it might become a limitation to translation of findings, because adaptive immune cells can play vital roles in promoting or inhibiting the progression of human cancers and the effects of certain cancer treatments (Gupta and Massagué, 2006; Kees and Egeblad, 2011; Spano et al., 2012).

Human and zebrafish genomes are 70% similar based on conservation of individual genes (Table 1), with several cancer-associated genes found in mammals but not in the zebrafish, including *BRCA1*, *p16* (*CDKN2A*), *BRCA1*, *LIF*, *OSM*, *IL6* (Howe et al., 2013) and *PML* (G.D. and J.N.B., unpublished observation). This presents several challenges when studying the functions of these ‘missing’ genes or the pathways in which they play a role; for example, senescence studies in the fish might prove to be challenging given the lack of a *p16* homolog, a major marker of senescence in mammals (Salama et al., 2014). Moreover, when foreign tissue and cells are introduced into fish, there is no guarantee that all of the molecular mechanisms linking the recipient and the xenograft tissue are completely conserved, which might impact interactions between host cells and the cancer xenograft. For example, human growth factors cannot support zebrafish hematopoiesis (Stachura et al., 2009). This issue is particularly relevant for tissues such as the lung, breast and prostate for which orthotopic sites do not exist in the fish. However, as discussed above, it might be possible to ‘add-back’ the required cells or growth signals to mitigate this problem during xenotransplantation, or to ‘humanize’ the fish by creating transgenic animals that express appropriate human growth factors, receptors and/or cytokines, as has been done in mice (Brehm et al., 2013).

**Table 1.**
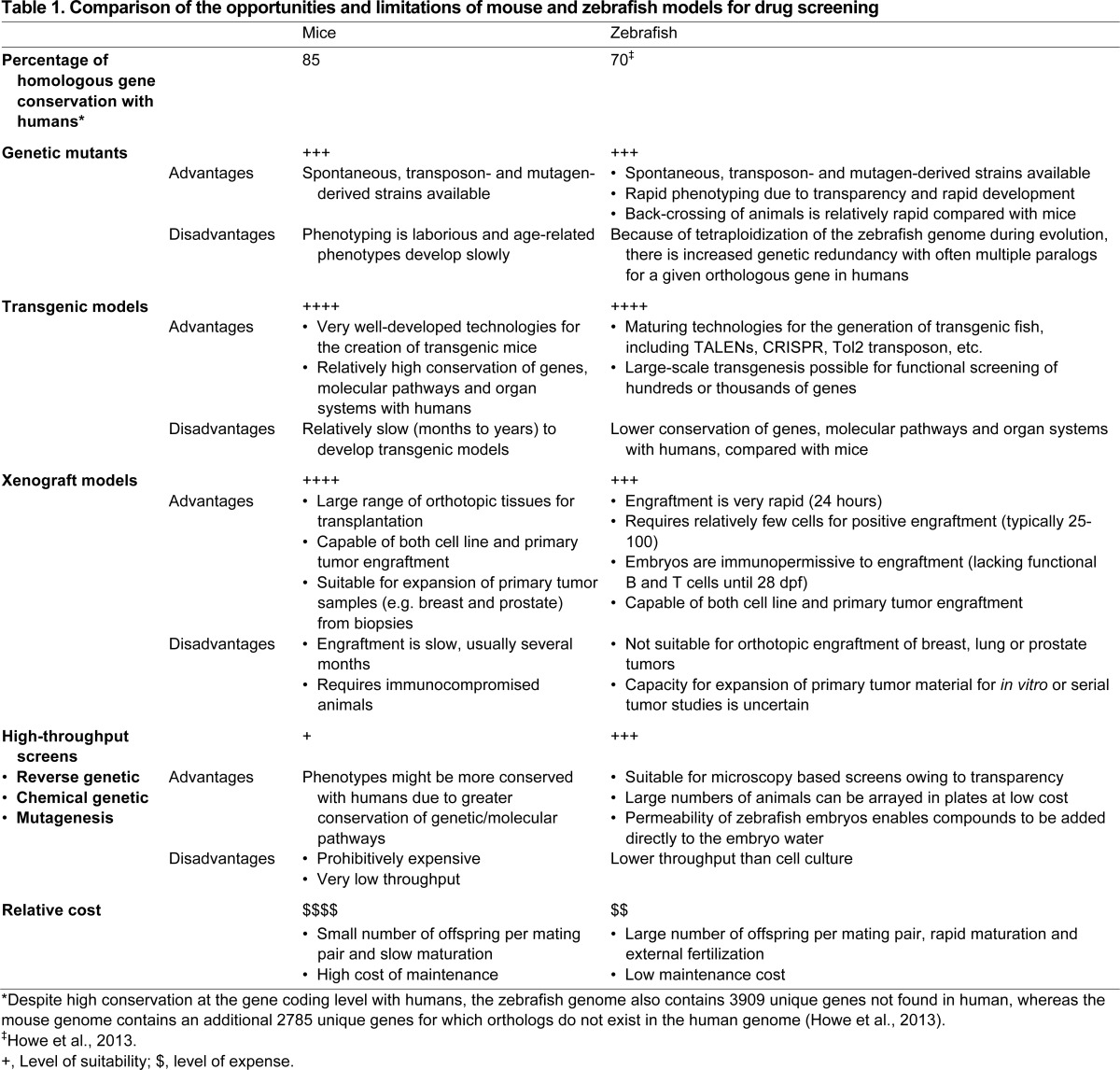
Comparison of the opportunities and limitations of mouse and zebrafish models for drug screening

## Clinical applications of the zebrafish xenotransplantation platform

Xenotransplantation approaches can be used for small-molecule screens using similar procedures to those employed by traditional zebrafish transgenic models, including arraying injected embryos into multi-well plates, with the added benefit of being able to measure cell proliferation and metastasis in the presence or absence of a drug.

A recent area of growth for zebrafish xenotransplantation in chemical screens is the progression towards enhanced experimental automation and rapid high-resolution imaging (Ghotra et al., 2012). Most xenotransplantation studies require manual injection of the human cells, which can be a time-consuming technical hurdle. Although a skilled researcher can inject a few hundred embryos in an hour, the use of automated methods would greatly expedite this process.

Efforts are now underway to apply this approach to patient-derived specimens. Primary human tumors, such as gastric cancers, prostate and primary leukemia samples, can be injected into embryos, where they proliferate and disseminate (Bansal et al., 2014; Marques et al., 2009; Pruvot et al., 2011) (V. L. Bentley, C.J.V., D. P. Corkery, J. B. Pinder, M. A. LeBlanc, K. Bedard et al., unpublished results). Murine xenotransplantation studies using primary patient samples can be very complex and relatively expensive. The total sample needed is several times greater in mice than in zebrafish xenografts (typically 100–200 cells are needed per zebrafish embryo), so zebrafish provide a key advantage when working with precious limited biopsy samples. Zebrafish engraftment can also take as little as 2–3 days, whereas, in a murine model, primary tissue grafts can take weeks to months, if engraftment occurs at all.

Given the speed of zebrafish xenotransplantation, it could be used as a predictive tool for patient responses to drug therapies. Transplanting malignant patient tissue or cells into hundreds of zebrafish embryos and monitoring the response to planned investigational drug treatments could yield valuable information on the most suitable drug to be administered for that particular patient. Evidence of a response to a small-molecule inhibitor might indicate effective targeting of an actionable genetic driver lesion, as we observed for a child with T-ALL who expressed a novel γ-secretase-responsive Notch mutation (Bentley et al., 2013) (V. L. Bentley, C.J.V., D. P. Corkery, J. B. Pinder, M. A. LeBlanc, K. Bedard et al., unpublished results). The ability of zebrafish xenotransplantation to provide such actionable personalized clinical information is promising and, with further validation, has the potential to find its way into future clinical trials.

The approach could be most readily adapted in leukemia, where the incorporation of targeted therapeutic strategies is becoming commonplace and diagnostic material is easily accessible. In contrast to current strategies where a targeted therapy could be randomly assigned to a patient who might or might not be screened to confirm the presence of a particular actionable genetic lesion, incorporation of the zebrafish xenograft platform could provide a more practical functional genomics readout with real time *in vivo* response data, which could be followed by genomic validation. Applicability to solid tumors is also feasible; however, here there is a need to overcome the challenges of limited availability of biopsy material and tumor heterogeneity (which could mean that the sample being engrafted is not representative of tumor genetics or behavior).

Diagnostic leukemia or solid tumor samples can also be used to predict responses to experimental therapies that might be considered in the event of refractory disease or relapse. Anecdotally, we have undertaken these studies, which could inform the selection of a particular Phase I/II trial. In most cases the eligibility for these studies is refractory or relapsed disease without specific evidence for the presence of a particular target or activity of a given pathway. Evidence of an efficacy signal from pre-screening available Phase I/II drug options using a zebrafish xenograft model could provide timely guidance in choosing the most promising therapy. Although encouraging in its ability to more specifically tailor therapy at relapse, this strategy could be limited by clonal evolution, which has the potential to significantly alter the genetics and response of a tumor from the time of diagnosis.

As is the case for other embryonic zebrafish chemical screens, in addition to evaluating drug efficacy, zebrafish xenotransplantation can help determine drug toxicity. By using xenografted embryos, the value of co-administration of a toxicity-mitigating agent can be studied in the context of ongoing primary drug efficacy. Using this approach, we confirmed that two novel compounds could prevent cardiotoxicity from anthracycline antibiotics without compromising cytotoxicity in an engrafted T-ALL cell line (Y. Liu, A. Asnani, L. Zou, V. L. Bentley, M. Yu, Y. Wang et al., unpublished results). Similarly, drug efficacy and neurotoxicity could be evaluated. The optical clarity of the zebrafish embryo enables clear visualization of neural structures and there is a battery of probes that label each distinct component of the central nervous system, facilitating the evaluation of drug-induced anatomic changes. Drugs of lower molecular weights more easily cross the embryonic blood-brain barrier, a factor to be taken into consideration when evaluating the impact on brain development of a compound given systemically. Finally, myelotoxicity is inherently evaluable in the zebrafish xenograft context. By conducting xenograft drug response studies that incorporate transgenic reporter lines that label neutrophils (Hsu et al., 2004; Renshaw et al., 2006), erythrocytes (Long et al., 1997) or thrombocytes (Lin et al., 2005), the impact of a drug on cell numbers can be visually determined and subsequently quantified using fluorescence-activated cell sorting.

## Conclusion

Transgenic and xenotransplantation approaches have long been used in a complementary manner in mice to evaluate responses of tumors to drugs. Transgenic approaches in zebrafish have become well established, with a growing acceptance and appreciation for the tremendous efficiency of high-throughput chemical screens in genetically modified embryos to provide new indications for the repurposing of available agents and reveal the potential efficacy of novel compounds. Although still in its infancy, the zebrafish xenotransplantation platform capitalizes on many of the advantages inherent in the zebrafish system, with additional opportunities in the evaluation of patient-derived samples, tumor-microenvironment interactions and the potential ability to inform treatment decisions in an actionable time frame. Like all techniques, zebrafish xenotransplantation is not without its limitations, and additional proof-of-principle studies are needed to conclusively show that features that have made the mouse xenotransplantation model the ‘gold standard’ for preclinical studies, such as the ability for orthotopic engraftment and serial transplantation, are equally robust in the fish (Table 1). Given current pressure on the pharmaceutical industry to use more cost-effective pre-screening tools for drug discovery, and the clinical demand for more targeted and personalized therapy, we predict that the popularity of the zebrafish xenotransplantation model will continue to increase and will provide a uniquely powerful tool to improve the outcome for patients with cancer.
